# The effect of body position on compartmental intra-abdominal pressure following liver transplantation

**DOI:** 10.1186/2110-5820-2-S1-S12

**Published:** 2012-07-05

**Authors:** Adrian B Cresswell, Wayel Jassem, Parthi Srinivasan, Andreas A Prachalias, Elizabeth Sizer, William Burnal, Georg Auzinger, Paolo Muiesan, Mohammed Rela, Nigel D Heaton, Matthew J Bowles, Julia A Wendon

**Affiliations:** 1 Liver Transplant Surgical Service and Liver Intensive Care Unit, Kings College London, Institute of Liver Studies, King's College Hospital, Denmark Hill, SE5 9RS, London, UK

**Keywords:** intra-abdominal pressure, abdominal compartment syndrome, intra-abdominal hypertension, liver transplantation, regional pressure variation

## Abstract

**Background:**

Current assumptions rely on intra-abdominal pressure (IAP) being uniform across the abdominal cavity. The abdominal contents are, however, a heterogeneous mix of solid, liquid and gas, and pressure transmission may not be uniform. The current study examines the upper and lower IAP following liver transplantation.

**Methods:**

IAP was measured directly via intra-peritoneal catheters placed at the liver and outside the bladder. Compartmental pressure data were recorded at 10-min intervals for up to 72 h following surgery, and the effect of intermittent posture change on compartmental pressures was also studied. Pelvic intra-peritoneal pressure was compared to intra-bladder pressure measured via a FoleyManometer.

**Results:**

A significant variation in upper and lower IAP of 18% was observed with a range of differences of 0 to 16 mmHg. A sustained difference in inter-compartmental pressure of 4 mmHg or more was present for 23% of the study time. Head-up positioning at 30° provided a protective effect on upper intra-abdominal pressure, resulting in a significant reduction in all patients. There was excellent agreement between intra-bladder and pelvic pressure.

**Conclusions:**

A clinically significant variation in inter-compartmental pressure exists following liver transplantation, which can be manipulated by changes to body position. The existence of regional pressure differences suggests that IAP monitoring at the bladder alone may under-diagnose intra-abdominal hypertension and abdominal compartment syndrome in these patients. The upper and lower abdomen may need to be considered as separate entities in certain conditions.

## Introduction

Interest in the measurement of intra-abdominal pressure (IAP) has grown steadily over the last decade and has been shown to be a significant problem within the general intensive care unit (ICU) population [1,2], with the deleterious effects of elevated IAP having been well described in numerous clinical studies and reviews [3-15]. The culmination of the recent increase in interest in this condition has been the creation, by an international panel of experts (The World Society on Abdominal Compartment Syndrome, WSACS, http://www.wsacs.org), of a consensus document for definitions [16] and suggested management guidelines [17].

Underpinning these recommendations, however, is a requirement for accurate and reproducible measurement of IAP with several studies having shown that there is no role for clinical estimation of IAP, either by palpation or measurement of abdominal perimeter [18-20].

Numerous techniques for the measurement of IAP by both direct and indirect methods have been described, with indirect approaches utilizing measurement of the pressure concealed within a hollow intra-abdominal viscus most usually the urinary bladder (intra-bladder pressure, IBP) [21] or stomach (intra-gastric pressure, IGP) [22]. Direct methods for measuring IAP have been employed exclusively in the experimental setting whereby the IAP is transduced directly from the peritoneal cavity via a catheter containing a continuous column of fluid [23], a balloon-tipped catheter [24] or via a laparoscopic gas insufflation system [25]. The application of such techniques is clearly limited by their invasiveness, and no advantage over indirect measurements has been demonstrated in terms of accuracy.

On the face of the available data, therefore, a non-invasive technique such as the IBP or IGP method would seem more attractive for routine clinical use. This, however, relies on two unproven assumptions regarding the transmission of pressure throughout the abdomen. The first assumption is that the bladder wall will act as a passive diaphragm for the transmission of pressure, and the second is that IAP is transmitted uniformly throughout the abdominal cavity such that the measured pressure at any one position will be reflected elsewhere in the cavity.

The second assumption relies on the contents of the intra-abdominal cavity, transmitting pressure as a single compartment, which, given the heterogeneous mix of contents, may not hold true. Such a regional variation in post-operative patients would have important implications both for the post-operative screening of IAP following surgery and for the potential of a localised effect on the regional organ systems that may not be manifested by the measurement of the relatively remote IBP. This concept would be synonymous with the poly-compartment syndrome which has previously been suggested to affect the head, thorax, abdomen [26] and extremities.

It has been shown that IAP can be influenced by body position with an increase in bladder pressure of up to 7.5 mmHg with a 45° positioning angle [27]. However, the effect of body position on the individual intra-abdominal compartment has not previously been described.

Liver transplantation was chosen for the study as a major intervention that has been shown to be associated with a significant incidence of intra-abdominal hypertension (IAH) in both our own unpublished data and in studies from other institutions [28]. The surgical procedure itself is relatively standardised and confined to a single intra-abdominal compartment, which makes comparisons between individual subjects easier and logically suggests that the chances of identifying a regional pressure phenomenon would be highest.

The two primary aims of the current study were to compare the IBP to that immediately outside within the intra-peritoneal pelvis and to establish whether there are any regional variations in IAP between the upper and lower abdominal compartments (upper intra-abdominal pressure, UIAP and lower intra-abdominal pressure, LIAP) following liver transplantation. A secondary endpoint was to examine the effect, if any, of body position on the compartmental pressures.

## Methods

Following approval of the study design by the local Research Ethics and Research & Development Committees, a total of 20 patients undergoing elective orthotopic transplantation were recruited, all of whom gave informed consent to take part in this study. All patients received cadaveric whole grafts and had not undergone liver transplantation previously. Data were collected during the subjects' stay on a 15-bed dedicated Liver Intensive Care Unit with aspects of post-operative care such as the administration of intra-venous fluids and the use of vaso-active agents, guided by established unit protocol. All subjects were nursed in a 30° head of bed position to minimise risk of respiratory complications with the exception of short periods of being laid flat in order to measure supine IAP. All were calm and comfortable at the time of measurement (Richmond agitation-sedation scale of 0).

For each patient, UIAP and LIAP were measured directly via catheters placed under the left lobe of the transplanted organ and in the pelvis at the time of operation (Minivac Drain, Unomedical, Worcestershire, UK). These catheters were connected, via a fluid column to an electronic pressure transducer with numeric and pressure trace displayed on the ICU monitor (Fukuda Denshi Co., Ltd, Tokyo, Japan). These catheters were used solely for measurement of IAP and not for drainage. Standard closed surgical drains were placed in the usual position to prevent accumulation of body fluids.

**Figure 1:**
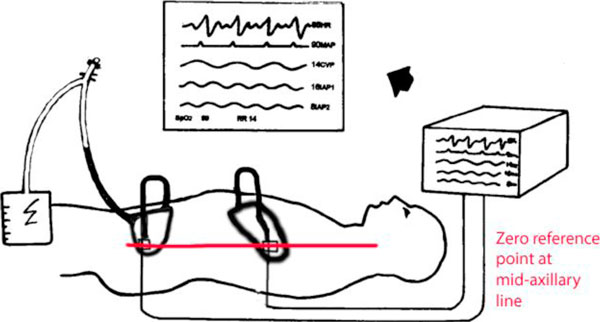
**Equipment set-up to transduce LIAP and UIAP along with FoleyManometer for measurement of IBP**.

The electronic transducers were fixed to the patient by sutures at a point corresponding to the internal position of the catheter tips on the upper and lower abdominal wall. A position that was found to correspond to the zero-reference point as suggested by the WSACS, of the mid-axillary line at the iliac crest when supine. The transducers were flushed and zeroed twice daily and after each patient position change. The measured dead space of the catheter was < 2 ml, and thus, a 4-ml flush with normal saline, from a sterile closed system, ensured a continuous column of fluid between the intra-peritoneal catheter tip and the transducer which was maintained between flushes by continuous low volume irrigation. The quality of the pressure waveform was checked hourly by the 'rapid oscillation test [21]', whereby rapid and repeated palpation of the abdominal wall at the level of the intra-peritoneal catheter tip was visible in real time on the ICU monitor's pressure trace (Figure 1).

Compartmental IAP was transduced continuously via this equipment, and the monitoring system recorded paired measurement of UIAP and LIAP at 10-min interval. The catheters were left in place for a maximum of 72 h or until the point of discharge from the Liver Intensive Care Unit, whichever came sooner.

Each patient was re-positioned to lie supine at 6-hour intervals (four times per day) in order to measure the supine compartmental pressures. The transducers were 're-zeroed' following each position change, and the pressure was allowed to equilibrate for 5 min prior to making each of these recordings.

**Figure 2:**
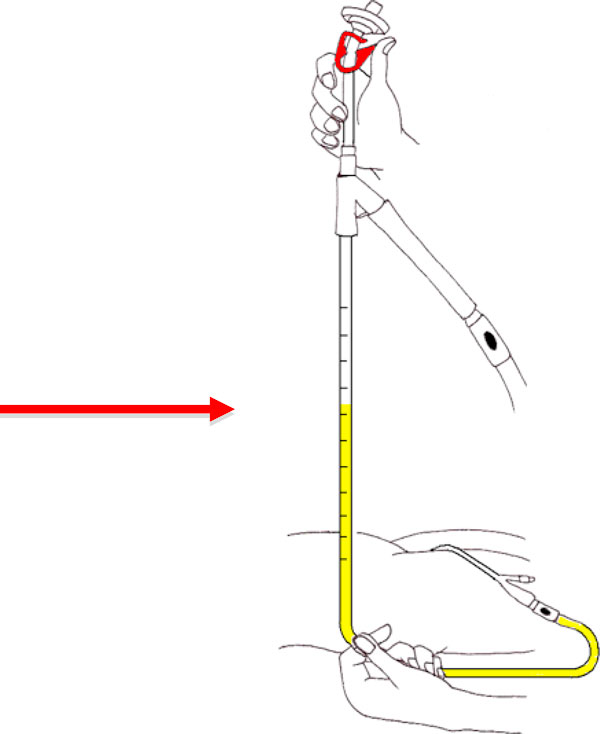
**Technique for using FoleyManometer for the measurement of intra-abdominal pressure**. IBP marked by arrow.

In addition to the direct pressure measurements, IBP was also recorded at 6-h intervals with the patient both in a 30° head up and supine positions using a FoleyManometer system (Holtech medical Company, Charlottenlund, Denmark), as shown in Figure 2.

### Statistical analysis

The data were recorded in a Microsoft Excel Spreadsheet (Microsoft, WA, USA) and analysed using SPSS v15 (Chicago, IL, USA) in accordance with the recommendations for data analysis published by the WSACS [29]. Data obtained at 6-hourly intervals (IBP, LIAP and UIAP at supine and 30° head of bed angles) were compared by means of a Bland and Altman analysis [30]. The coefficient of variance of IAP was defined as the standard deviation of IAP divided by the mean IAP. Percentage error of the measurement was defined as twice the precision divided by the mean IAP. The normality of distribution of the continuous pressure recordings was tested using a Kolmogorov-Smirnov test, and being parametric and normally distributed, means were compared using a paired *t *test.

Following professional statistical advice and in order to perform both within and between individual comparisons of the difference in compartmental pressures in subjects with differing baseline IAP, the difference between the two compartmental recordings was converted to a percentage of the mean of both compartments (*Difference *÷ (*mean of UIAP *+ *LIAP*) × 100). This eliminated the effect of the underlying baseline IAP and inter-individual variations. For the same reason, the trend in compartmental pressure over time was expressed as the difference in each subsequent pressure recording over the initial IAP. The differences in compartmental pressures over time were normally distributed and, therefore, compared by linear regression. For the purpose of reporting, a difference of 4 mmHg or greater between the compartments was considered to be clinically significant.

## Results

### Comparison of direct and indirect measurement of lower intra-abdominal pressure

**Figure 3:**
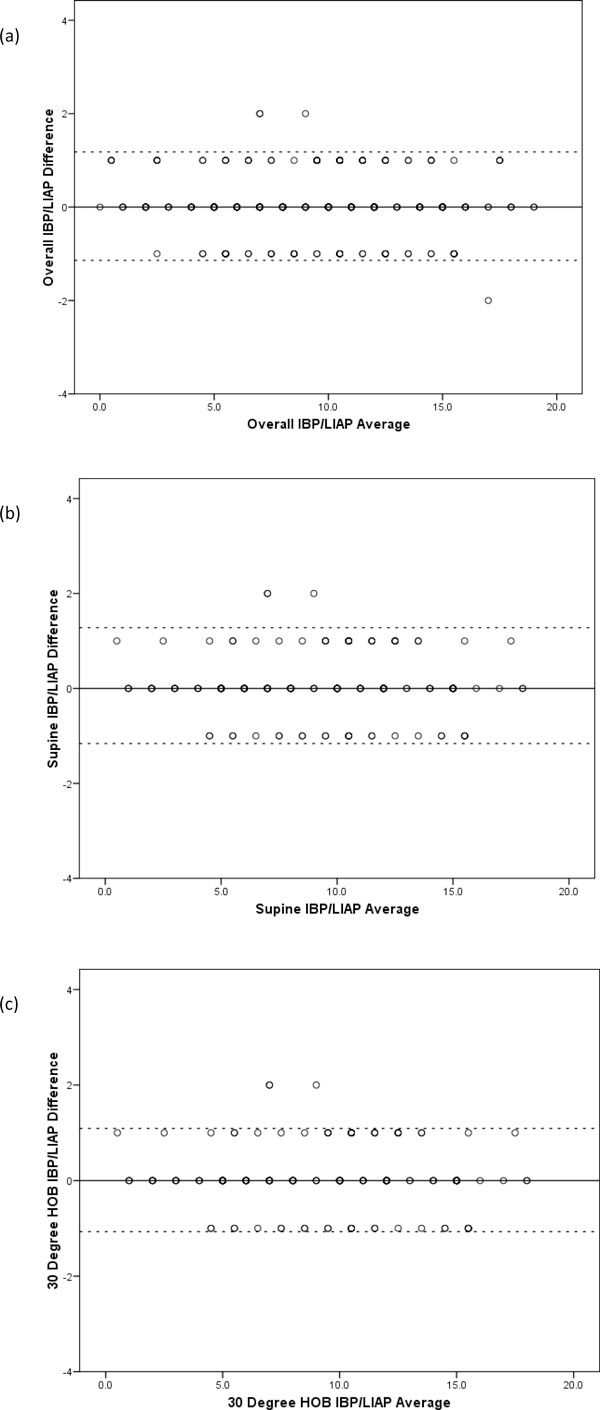
**Bland and Altman plots comparing intra-bladder pressure (IBP) and lower intra-abdominal pressure (LIAP)**. (**a**) Bland and Altman plot to compare IBP and LIAP with a supine body position and a head of bed position of 30°. (**b**) Bland and Altman plot to compare IBP and LIAP with a supine body position.(**c**) Bland and Altman plot to compare IBP and LIAP with a head of bed position of 30°. Lower level of agreement (LLA) and upper level of agreement (ULA) marked by dotted lines.

There was no clinically relevant difference between the mean measurements made via the pelvic transducer and the foley manometer. The Bland and Altman plot (Figure 3a, b, c) confirmed excellent agreement between the two measures in all body positions, with a calculated bias and precision of -0.06 and 0.6 when supine and 0.006 and 0.5 at 30°. Full details of the two measurements are given in Table 1.

**Table 1:** Comparison of IBP, LIAP and UIAP

Comparisons		Number	Mean IAP	Range IAP	COVA IAP	***r***^a^	*p*	Bias	Precision	LLA	ULA	% Error
**IBP vs LIAP**	All	338	9.43	0.0 to 19.0	43.6	0.99	< 0.001	0.03	0.59	-1.14	1.18	13
	Supine	169	9.23	0.5 to 18.0	43.7	0.99	< 0.001	0.06	0.62	-1.16	1.28	13
	30° HOB	169	9.63	0.0 to 19.0	43.6	0.99	< 0.001	-0.01	0.55	-1.07	1.09	11
**IBP vs UIAP**	All	338	10.07	1.5 to 19.5	39.7	0.66	< 0.001	-1.25	3.63	-8.36	5.86	72
	Supine	169	10.49	2.5 to 19.5	38.9	0.70	< 0.001	-2.46	3.52	-9.36	4.44	67
	30° HOB	169	9.65	1.5 to 18.5	39.9	0.68	< 0.001	-0.05	3.34	-6.60	6.5	69
**LIAP vs UIAP**	All	338	10.06	1.5 to 19.5	39.8	0.66	< 0.001	-1.28	3.63	-7.31	4.75	72
	Supine	169	10.46	2.5 to 19.5	39.3	0.70	< 0.001	-2.51	3.47	-9.31	4.29	66
	30° HOB	169	9.66	1.5 to 18.0	40.1	0.68	< 0.001	-0.05	3.36	-6.64	6.54	70

The patient positioned supine and in a 30° head of bed angle. IBP, intra-bladder pressure; LIAP, lower intra-abdominal pressure; UIAP, upper intra-abdominal pressure; HOB, head of bed, IAP, intra-abdominal pressure; COVA; coefficient of variance; LLA, Lower level of agreement; ULA, Upper level of agreement. ^a^*r *= Pearson product-moment correlation coefficient.

### Compartmental pressure measurements

**Figure 4:**
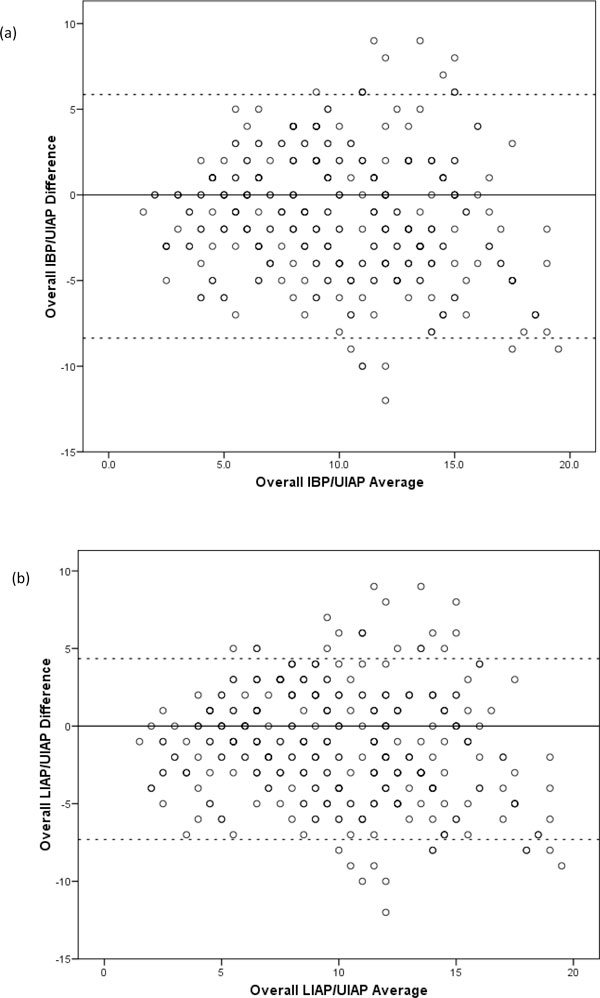
**Intra-bladder pressure (IBP) and upper intra-abdominal pressure (UIAP), and lower intra-abdominal pressure (LIAP) and UIAP**. (**a**) Bland and Altman plot to compare IBP and UIAP with a supine body position and a head of bed position of 30°. (**b**) Bland and Altman plot to compare LIAP and UIAP with a supine body position and a head of bed position of 30°. LLA and ULA marked by dotted lines.

A total of 169 synchronous measurements of IBP, LIAP and UIAP were made to obtain compartmental pressure with subjects in a supine and 30° head of bed position at 6-h intervals. In contrast to the excellent agreement between IBP and LIAP, comparisons of both IBP and UIAP, and LIAP and UIAP revealed very poor agreement with a high measured bias, precision and percentage error (Table 1 and Figure 4a, b). Parameters for these comparisons fell well outside the thresholds for agreement stated by the WSACS [29].

The mean UIAP when supine was 11.7 mmHg, which was reduced to 9.6 mmHg with 30° head of bed positioning (*p *< 0.001). Mean LIAP was 9.2 mmHg when supine and increased to 9.6 mmHg with 30° head of bed (*p *< 0.001).

The increase in UIAP with a move to a supine position was observed in all patients, irrespective of which compartment contained the higher pressure. The observed magnitude of change in mean UIAP was not different between subjects exhibiting a raised IAP (> 12 mmHg) and those with a normal IAP (2.4 and 1.8 mmHg change, respectively; *p *= 0.5). Similarly, although there was a suggestion that subjects with higher upper than lower compartmental pressures exhibited a larger change in UIAP when moving to a supine position (2.4 and 1.1 mmHg change, respectively), this difference was not statistically significant (*p *= 0.9).

### Continuous pressure measurements

A total of 5,980 automated-paired pressure measurements of direct LIAP and UIAP were recorded with an average of 299 per patient (range 212 to 461). Of the 20 subjects, 12 revealed a higher mean pressure within the UIAP than the LIAP compartment, with the greatest mean pressure difference for an individual being 5.3 mmHg.

When analysed as a whole and as sub-groups with either higher UIAP or higher LIAP, the difference between the compartmental pressures was highly statistically significant (*p *< 0.001, *p *< 0.001 and *p *< 0.004, respectively). The range of differences between compartmental pressures in the two groups also differed with those exhibiting a higher UIAP having a broader range (0 to 16 mmHg) than those with a higher LIAP (0 to 12 mmHg). The mean pressures observed in each compartment for the subjects as a whole and for the two sub-groups are displayed in Table 2.

**Table 2:** Mean continuous pressures observed in each compartment, split according to highest average compartment pressure

Subject group	Mean compartmental IAP (mmHg, SD)		Mean difference between compartments (SD)	**Mean percentage difference between compartments**^a^
			
	UIAP	LIAP		
**Higher**	10.5	8.3	2.2	23.4%
**UIAP**	(4.6)	(4.5)	(2.4)	
**Higher**	8.6	10.9	2.3	23.6%
**LIAP**	(3.8)	(5.6)	(3.2)	
**Overall**	9.7	9.5	0.3	3.1%
	(4.2)	(4.6)	(4.1)	

UIAP, upper intra-abdominal pressure; LIAP, lower intra-abdominal pressure; SD, Standard Deviation. ^a^Mean percentage difference between compartments = mean difference between compartments/(mean UIAP + mean LIAP/2) × 100.

The mean difference between the two compartments was similar, whether it was the upper or lower compartment that contained the higher pressure. Expressed as a percentage of the mean of the two compartments, this equated to a clinically significant 23.4% difference when UIAP was highest, and 23.6% when LIAP was highest.

Individual analysis of each subject's data confirmed the significant difference (*p *< 0.001) between compartments for all but two patients. In two individuals, the compartmental pressures did not differ significantly (*p *= 0.349 and 0.122, respectively); however, the mean IAPs for both patients and in both compartments fell within normal safe limits (7.1 and 7.3 mmHg, and 11.4 and 12.0 mmHg, respectively).

Nine subjects displayed a continuous pressure > 12 mmHg in one or other compartment for greater than 1 h. Of these, five had higher mean UIAP, and four had higher LIAP. There was no difference between the mean difference in compartmental pressures in subjects with a sustained pressure of > 12 mmHg compared to those without (2.3 and 2.1 mmHg, respectively; *p *= 0.772).

In the higher UIAP group, a clinically significant difference of 4 mmHg or more between compartmental pressures was observed during an average of 23% of the study period. This proportion was higher in the higher LIAP group at 37% of the study duration; however, these differences were not statistically significant (*p *= 0.666). The direction of change in compartmental IAP over time correlated positively such that an overall upward trend in UIAP was accompanied by an upward trend in LIAP (*r^2 ^*= 0.582, *p *< 0.001, *n *= 5,960).

## Discussion

The recognition and treatment of IAH and the abdominal compartment syndrome (ACS) are clearly reliant on an accurate and reliable system for the measurement of IAP. The technique for IBP measurement has undergone much refinement over the last decade [21] and has now been presented, by an international panel of experts, as the gold standard for intra-abdominal pressure measurement [17]. In addition to the effects of gravity and sheer stress [31], the value of bladder pressure relies on two key assumptions which have been widely accepted without direct evidence of their validity.

The first assumption is that the bladder wall will act as a passive diaphragm to the transmission of pressure, and therefore, the pressure measured within the urinary bladder will accurately reflect the pressure immediately outside within the peritoneal cavity. Several studies, in both animal and human models, have shown good agreement between directly and indirectly measured intra-abdominal pressure [22,23,32,33]. All of these studies, however, have measured direct IAP at a site distant to the urinary bladder and followed artificial elevation of IAP by means of either saline or gas insufflation, or by insertion of a mechanical prosthesis. Our data is the first to directly compare the pressure measured at the intra-vesical and intra-peritoneal sides of the bladder wall and confirms that the pressure measured within the urinary bladder demonstrates excellent agreement with the pressure to be measured within the pelvic peritoneal cavity.

The second assumption relates to the mechanical properties of the peritoneal contents. It has been suggested that the abdominal contents are primarily fluid in composition and, therefore, that pressure transmission can be expected to follow Pascal's law such that measurement of the IAP at any point will reflect the pressure contained within the entire abdominal cavity [21]. In reality, however, the abdominal contents remain a heterogeneous mix of solid, liquid and gaseous components with the exact composition influenced by several disease processes such as paralytic ileus, visceral oedema, or the presence of ascites. Pressure transmission characteristics are, therefore, likely to be rather more complex.

### Regional IAP

The implications of a regional ACS are profound with the gold-standard technique for pressure measurement occurring at the lowest point in the abdominal cavity, whilst the organs that have been shown to be most susceptible to raised IAP all lie in the upper abdomen. Separate studies have all clearly shown the deleterious effects of raised IAP on the splanchnic circulation [11-13,34,35], cardiac [8,36], respiratory [9,37,38], renal [5,32,39] and neurological [40,41] functions in both human and animal models.

The possibility of a regional variation between the upper and lower IAP was identified, but not explored in detail in 1994 [22]. In this study, IGP was measured in nine patients undergoing laparoscopic cholecystectomy at a variety of different insufflation pressures. The study was designed to validate the measurement of IGP against the pneumoperitoneum but also showed that IGP could also be up to 4 mmHg higher or 3 mmHg lower than the measured IBP. A further small study has identified differences in gastric and bladder pressure in two patients within a general ICU population [42] and suggested that such a variation could provide clues as to any underlying pathophysiological process.

Our study is the largest to compare the two compartmental pressures within a clinical setting, without artificial manipulation of IAP. In keeping with the above study, we showed a significant difference between compartmental pressures but with a much broader and more clinically significant range of variation of up to 16 mmHg and a mean difference between the compartments of around 20% which equates to a maximal inter-compartmental mean difference of 5.3 mmHg.

Clearly, such a magnitude of variation, coupled with the observation that compartmental pressures were seen to vary by 4 mmHg or more for an average of 23% of the time, means that relying on the measurement of one compartmental pressure only may lead to a significantly elevated pressure in the other compartment being missed. The positive relationship that we have demonstrated between compartmental pressures should mandate separate measurement of UIAP in patients in whom the IBP is adopting an upward trend.

It was interesting to observe that the range of variation in inter-compartmental pressure was greater in those patients concealing a higher UIAP, and this may be related to the previous data which suggest that upper abdominal incisions result in measurable changes to abdominal wall contractile properties which may contribute to the generation of a locally raised IAP [43].

### Body position and regional IAP

Previous clinical studies have considered the influence of patient positioning on IAP. In the largest [44], a multi-centre study of 132 ventilated patients, the mean difference between supine and 30° IBP was 3.7 mmHg with a range of 3.4 to 4.0 mmHg. The largest reported difference in positional pressures was seen in a study of 37 patients at a range of bed positions between 0 and 45° [27]. It was found that IBP increased with head-up tilt with a mean increase of 5 mmHg at 30°, and 7.4 mmHg at 45°.

Our data have also demonstrated a statistically significant increase in the IBP with head-up positioning to 30°. This was, however, a far smaller increase of just 0.43 mmHg rather than the 5 mmHg seen in the above study. This would lend support to the theory that LIAP will increase as the result of a more upright posture [33]. The most likely explanation for this is that an erect posture leads to an increase in the hydrostatic weight exerted by the abdominal organs and body habitus pressing downwards on the bladder much in the same manner as increasing the height of a standing column of fluid would increase the measurable pressure at the bottom of the column.

A more interesting observation in our own data, however, is the fact that despite accurate re-zeroing of a patient mounted transducer UIAP was significantly increased in the supine position compared to a 30° head-up tilt. The reason for this observation remains unclear but may be related to the re-positioning of the more mobile hollow abdominal viscera along with both their fluid contents and any free intra-peritoneal fluid with a more upright posture. This observation would suggest that a simple change in posture could provide a clinically significant improvement in the UIAP, which in turn, may improve hepatic, renal and splanchnic blood flow. Such positive effects on organ perfusion would need to be demonstrated by further specific studies; however, it does raise the possibility that a head-up position may be advantageous for reasons other than simple ventilatory mechanics. It is also particularly encouraging to note that a larger reduction in UIAP can be expected in those patients with a higher upper, rather than lower, baseline intra-abdominal pressure. The lack of collection of other body anthropomorphic data to further examine these two groups is accepted to be an unfortunate limitation of the study.

### Clinical application

The fact that it was impossible to predict which of the two compartments would conceal the higher pressure suggests that, for this subgroup of patients, dual compartmental pressure monitoring may be required based upon the clinical condition of the patient. It remains unclear, however, whether the observed variation in inter-compartmental pressure is specific to the procedure of liver transplantation, or whether the findings could be generalised to all upper abdominal surgery, local inflammatory conditions such as severe acute pancreatitis, or indeed the measurement of IAP in general. It is also a shortcoming that various anthropomorphic data and details of illness severity scores were not collected, as these have been shown to impact on baseline IAP.

Further study with a larger sample size will be required to elucidate the relationship between the location of the higher compartmental pressure, the magnitude of variation in compartmental pressure and the duration for which there is a significant difference between compartments with clinical outcome. Such a study, with higher numbers, may be facilitated by the recent introduction of a commercially available non-invasive device for the measurement of IGP (CiMON, Pulsion Medical Systems, Munich, Germany). It would also be extremely interesting to measure the retroperitoneal compartmental pressure within the upper abdomen which very much contains the 'anatomical terminus' for the arrival and departure of the abdominal blood supply, as well as the kidneys themselves.

## Conclusion

It remains to be seen and further research is certainly required to discover whether the observed effects are specific to patients undergoing liver transplantation and to define any effects on clinical outcome. The current data do, however, demonstrate a significant variation in regional IAP within the study group. It may be well that we need to consider regional IAP in more detail and consider the different abdominal compartments, including the retroperitoneum, as more distinct entities, and patient positioning may prove a useful utility for optimising compartmental pressures and perfusion.

## Abbreviations

ACS: abdominal compartment syndrome; IAH: intra-abdominal hypertension; IAP: intra-abdominal pressure; IBP: intra-bladder pressure; ICU: intensive care unit; IGP: intra-gastric pressure; LIAP: lower intra-abdominal pressure; UIAP: upper intra-abdominal pressure; WSACS: World Society on Abdominal Compartment Syndrome.

## Consent

Written informed consent was obtained from the patient for publication of this case report and accompanying images. A copy of the written consent is available for review by the Editor-in-Chief of this journal.

## Competing interests

Dr Julia Wendon is a member of the medical advisory board of Pulsion Medical Systems, who have sponsored the processing fees of this submission. The authors declare that they have no other conflicts of interests.

## Authors' contributions

ABC conceived of the idea, designed the study, arranged ethical approval, conducted the study, collected the data, analysed the data and wrote the manuscript. JAW and MJB reviewed the study design, assisted with analysis and reviewed the manuscript. WJ, PS, AAP, ES, WB, GA, PM, MR and NDH assisted in the insertion and the day-to-day care of the experimental pressure catheters.

## Authors' information

ABC is now a hepatopancreatobiliary (HPB) surgeon at the Basingstoke Hepatobiliary Unit, UK. WJ, PS, AAP, MR and NDH are HPB and liver transplant surgeons at King's College Hospital, UK. JAW, ES, WB and GA are liver intensivists at King's College Hospital, UK. MJB is an HPB surgeon at Derriford Hospital, UK but undertook this work whilst a HPB and transplant surgeon at Kings College Hospital.
